# Chikungunya Virus Outbreak, Dominica, 2014

**DOI:** 10.3201/eid2105.141813

**Published:** 2015-05

**Authors:** Shalauddin Ahmed, Lorraine Francis, R. Paul Ricketts, Trudy Christian, Karen Polson-Edwards, Babatunde Olowokure

**Affiliations:** Ministry of Health and Environment, Roseau, Dominica (S. Ahmed, R.P. Ricketts, T. Christian);; Caribbean Public Health Agency, Port-of-Spain, Trinidad (L. Francis, K. Polson-Edwards, B. Olowokure)

**Keywords:** Chikungunya, virus, vectorborne infections, mosquito, Dominica, viruses, outbreaks

**To the Editor:** Chikungunya is a dengue-like mosquitoborne viral disease that has caused outbreaks in Africa, Asia, and the Pacific Islands (*1*). St. Martin reported the first documented occurrence of autochthonous transmission of chikungunya in the Caribbean islands in December 2013 (*2*). Dominica reported its first case on January 17, 2014 (*3*). This report describes the outbreak of chikungunya in Dominica through July 12, 2014.

Cases were characterized by using guidelines issued by the Centers for Disease Control and Prevention (CDC) and the Pan American Health Organization (*4*). Surveillance of chikungunya cases began on January 16, 2014, and data were collected on patients’ age, sex, residence, date of illness onset, clinical features, and travel history.

The virus was detected at the Caribbean Public Health Agency (CARPHA) laboratory in Trinidad by using a real-time PCR (rPCR) developed by CDC; some testing was also done at CDC’s Arboviral Diseases Branch in Fort Collins, Colorado, USA, by using an IgM ELISA and a plaque-reduction neutralization test, as appropriate. All suspected infections were laboratory confirmed through April 30, 2014, when community transmission was established. Thereafter, testing was done only for patients hospitalized >48 hours, women in their third trimester of pregnancy, patients who died, or patients thought to be infected and coming from geographic areas where chikungunya transmission was not yet established..

During December 15, 2013–July 12, 2014, a total of 3,559 chikungunya cases were reported in Dominica, of which 141 were confirmed by laboratory testing (134 [95%] by rPCR, 7 [5%] by serologic methods). The remaining 3,418 patients were considered infected (Figure), indicating an overall attack rate of 5% (on the basis of Dominica’s census population for 2011, 71,293). Retrospective investigation showed that the 2 index patients experienced onset of illness during the week beginning December 15, 2013, and 1 of the patients had recently traveled from St. Martin. The 2 patients were unrelated and resided far apart.

**Figure:**
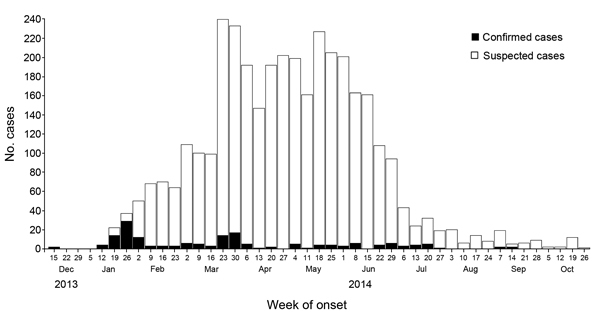
Confirmed and suspected chikungunya cases, by week of illness onset, Dominica, December 15, 2013–October 26, 2014.

Of the 141 confirmed patients, 78 (55%) were female and 60 (43%) were male; data on sex was unavailable for 3 patients. Mean age of the patients was 34 years (range 13 days–87 years; median 30 years). Thirty (21%) of the patients were children <9 years of age; 76 (55%) were 19–49 years of age. Most patients experienced fever (95%) and arthralgia (72%), and 21% of patients experienced rash. No deaths associated with chikungunya infection in Dominica were reported during the study period.

Across all age groups, more patients were female than male, as reported in previous outbreaks (*5*,*6*). This trend may suggest that, compared with men and boys, women and girls have greater health-seeking behaviors, greater levels of skin exposure, and potentially greater exposure due to peridomestic activities (*7*).

In this study, a disproportionate number of patients were <9 years of age, unlike findings for chikungunya outbreaks in Indonesia and Réunion Island, where children <9 years of age were least affected (*7*). Of all confirmed patients, 55% were 19–49 years of age, suggesting that the outbreak had economic effects because workplace productivity may substantially decrease if disease sequelae (e.g., arthralgia and arthritis) cause those affected to take time off from work.

Genotypic sequencing identified the Asian genotype of chikungunya as the strain currently circulating in the Caribbean (*8*,*9*). The East/Central/South African genotype was responsible for the Réunion Island outbreak, and an overall attack rate of 35% was reported after retrospective and active case detection (*6*). Differences in transmission and pathogenicity between genotypes require further investigation.

In response to the Dominica outbreak, a risk communication plan was developed and implemented on January 17, 2014, and consisted of 2 phases: an onset emergency phase and a control phase. Both phases targeted audiences through audio, print, and social media. To control and reduce the mosquito population in and around the homes of chikungunya patients, vector control activities (i.e., source reduction, application of larvicides, and fogging) were intensified with assistance from CARPHA and Yale University, New Haven, CT, USA. In addition, CARPHA and the Pan American Health Organization arranged for delivery of insecticide-treated bed nets for use in hospitals and other health care settings.

Although the introduction of chikungunya into the Caribbean islands may have been anticipated because of the broad distribution of the *Aedes aegypti* mosquito vector and suitable climatic conditions, our findings show that this outbreak could not be prevented. The continuing geographic spread of the disease emphasizes the ongoing challenge posed by mosquitoborne viral infections resulting from globalization and indicates a need for innovative prevention and control strategies.
